# Improving Anchoring Vignette Methodology in Health Surveys with Image Vignettes

**DOI:** 10.12758/mda.2022.02

**Published:** 2022

**Authors:** Mengyao Hu, Sunghee Lee, Hongwei Xu, Roberto Melipillán, Jacqui Smith, Arie Kapteyn

**Affiliations:** 1 University of Michigan-Ann Arbor; 2 Queens College, New York; 3 Universidad Del Desarrollo, Chile; 4 University of Southern California

**Keywords:** Differential item functioning, Anchoring vignettes, Image vignettes, Cross-cultural comparisons, Self-assessments of health

## Abstract

The anchoring vignette method is designed to improve comparisons across population groups and adjust for differential item functioning (DIF). Vignette questions are brief descriptions of hypothetical persons for respondents to rate. Although this method has been adopted widely in health surveys, there remain challenges. In particular, vignettes are complex, increasing survey time and respondent burden. Further, the assumptions underlying this method are often violated. To overcome such challenges, this paper introduces an innovative technique, namely image anchoring vignettes, conveying vignette information with varying health levels in images. We conducted a cross-cultural experimental study to examine the performance of image and standard text vignettes in terms of response time, how well they satisfy the assumptions, and their DIF-adjusting quality using a confirmatory factor analysis. The study revealed that respondents can better differentiate the intensity levels of the three vignettes in the image vignette condition, compared to text vignettes. Response consistency assumption appears to be better satisfied for image vignettes than text vignettes. Using well-designed image vignettes greatly reduces survey time without losing the DIF-adjustment quality, indicating the potential of image vignettes to improve overall efficiencies of the anchoring vignette method. Improving vignette equivalence (i.e., minimizing different interpretations of vignettes by different groups), remains a challenge for both text and image vignettes. This study generates new insights into the design and use of image anchoring vignettes.

Self-assessed questions on health are good predictors for mortality and morbidity (Idler & Benyamini, 1997; DeSalvo et al., 2005). Self-assessment health questions often use Likert-type rating scales to measure respondents’ attitudes, knowledge, perceptions, and behavior (Krosnick & Abelson, 1992; Lee, Jones, Mineyama, & Zhang, 2002). Ideally, responses obtained from these questions reflect only respondents’ true state. This, however, is not always the case. In fact, answers to self-assessment questions reflect both respondents’ true state and how they use the scales, a phenomenon known as response-category differential item functioning (DIF) (King, Murray, Salomon, & Tandon, 2004; King & Wand, 2007). As described in King and Wand (2007), DIF refers to situations when respondents from different backgrounds map the same state onto the scales in different ways.

Figure 1 (adapted from Hu, Lee, & Xu, 2018) illustrates a cross-cultural study example of DIF to a self-assessed pain question on an ordinal response scale from “None” to “Extreme”. In this example, cultural groups, A and B, use different cut points for a given response category. Assume that two respondents, one from A and one from B, have the same true pain level, both falling on the vertical dashed line. Despite their identical pain levels, the respondent from A will select “Mild,” and the respondent from B will choose “Moderate”. If this DIF is not accounted for, simple between-culture comparisons will erroneously conclude that the Culture B respondent experiences a higher level of pain (Hu et al., 2018).

An adjustment method for such DIF issues is to use anchoring vignettes (AV), which have been used in multiple national and international health surveys including the Health and Retirement Study (HRS) and the Survey of Health, Ageing and Retirement in Europe (SHARE). The AV approach typically involves two components: a self-assessment question and (typically multiple) anchoring vignette questions. First, respondents are asked to report their own status. For example, in a health survey, a typical self-assessed pain question is: Overall, in the last 30 days, how much pain or bodily aches did you have? The second component consists of vignette questions, each in a few sentences describing a hypothetical person’s situation related to the construct measured, and respondents are asked to rate the vignette person. For example, a vignette used in HRS asks, “*Paul has a headache once a month that is relieved after taking a pill. During the headache he can carry on with his day-to-day affairs. Overall, in the last 30 days, how much of a problem did Paul have with bodily aches or pains?*”. Usually, more than one vignette question describing varying intensity levels of the measured construct (e.g., low, moderate, and high levels) are asked (see Appendix 1). The vignette ratings can serve as benchmarks for the actual unobserved self-assessed pain level that researchers intend to measure.

The successful use of anchoring vignettes depends on two key assumptions: response consistency (RC) and vignette equivalence (VE). RC requires respondents to rate vignette persons in the same way as they would rate themselves (King et al., 2004). VE assumes that vignette descriptions are perceived similarly across respondents (King et al., 2004), essentially requiring vignettes to provide the same stimuli across respondents.

## Promises and Pitfalls of the Current Anchoring Vignette Approach

Anchoring vignettes (AV) have been reported in many studies as a promising tool to correct for DIF (e.g., Mojtabai, 2015; Murray et al., 2002). Despite its promise, studies of the effectiveness of the standard AV (which rely on verbal descriptions of the vignette persons) have yielded mixed results. While some studies have found that text vignettes can effectively correct for DIF (Dowd & Todd, 2011; Van Soest, Delaney, Harmon, Kapteyn, & Smith, 2011), other studies have reported that text vignettes do not necessarily provide comparable results among population groups (e.g., Grol-Prokopczyk et al., 2015). Previous studies have also shown that RC and VE assumptions can be violated in different domains (Bolt, Lu, & Kim, 2014; Ferrer-i-Carbonell, Van Praag, & Theodossiou, 2011; Kapteyn, Smith, Van Soest, & Vonková, 2011; Rice, Robone, & Smith, 2012).

The assumption violations are likely due to several practical challenges related to the AV design [see also Hu and colleagues (2018)]. The first and most obvious challenge concerns question difficulty (Hopkins & King, 2010). Unlike typical survey questions that ask respondents to rate their own status, AV require respondents to imagine hypothetical persons based on verbal descriptions and to shift their focus from themselves to rate the status of these imagined hypothetical persons, placing greater cognitive burden on respondents. The second challenge is a substantial increase in survey time. Given that vignettes are designed to describe hypothetical situations, one single vignette often contains much more text than other typical survey questions (Hu and Lee, 2016). In addition, because usually more than one vignette is used per domain (e.g., pain), the use of AV may require a non-trivial amount of response time (Hirve et al., 2013; Hopkins & King, 2010; King et al., 2004). Third, the use of AV in cross-cultural research raises yet another issue with text vignettes: measurement inequivalence, where respondents with different cultural background may understand vignette descriptions in systematically different ways. One source that can lead to measurement inequivalence is questionnaire translation. Poor translation can directly influence respondents’ interpretation of the vignettes, leading to violation of the VE assumption. Another critical challenge is the specific content to include in vignette descriptions. As acknowledged by Kapteyn et al. (2011), it is difficult to write vignette descriptions that are as “comprehensive” as what respondents know about their own state (Kapteyn et al., 2011). This indicates that respondents may rate themselves using criteria different from those they use for vignettes, resulting in violation of the RC assumption. VE can also be violated if respondents interpret the vignette descriptions in different ways. The potential for this problem is even greater in cross-cultural research where the challenges of designing equivalent and comparable vignettes are increased.

Although previous literature has greatly emphasized the importance of the design and pretesting of text AV, no clear design guidelines have been established to address the above limitations and practical challenges.

## Image Anchoring Vignettes

As a potential remedy to the limitations of text AV, we propose in this study to use visual AV with well-designed and carefully-selected images, i.e., image vignettes. With the technical development of internet, image vignettes have gained increasing popularity in survey research, especially in studying attitudes and sensitive questions (Naylor et al., 2014; Groot et al., 2020). To the best of our knowledge, this study is the first research that incorporates visual methodology with AV techniques.

Mechanisms of information processing of visual vs. verbal stimuli have been discussed in previous studies but there are no consensus conclusions. Some studies report similar processing of visual and verbal information in “a functional unitary system that is directly accessed by both visual objects and words” (Caramazza, 1996). In contrast, some other studies have shown that visual and verbal information are processed differently and “creating separate semantic representations” (Glaser, 1992; Glaser & Glaser, 1989; Schlochtermeier et al., 2013). For example, information processing of images is reported to be connected to activation of the right brain hemisphere (Grady et al., 1998; Naspetti et al., 2016), and activation of the left hemisphere is found to be associated with text information processing (Sevostianov et al., 2002). Despite the inconclusive results of the mechanisms of information processing, a common finding reported in previous studies is the “processing superiority” of images as compared to text information (Azizian et al., 2006, Schlochtermeier et al., 2013). As reported in Schlochtermeier et al. (2013), images lead to faster and a more direct access to meaning. In comparison, texts require “additional translational activity at the representational level” to access the semantic system (Schlochtermeier et al., 2013).

Given the reported processing superiority of image processing, the image AV strategy may lead to several potential advantages. First, images may require less cognitive effort to process than do text descriptions. Compared to texts, images are processed in a quicker and more automatic way, allowing respondents to form more “direct” connections between images and their meaning (Luna & Peracchio, 2003; Paivio, 2013; Townsend & Kahn, 2014). In the case of AV (which require imagining hypothetical persons), the use of images is advantageous for both low-literacy respondents and those who are unable to create mental images based on text vignettes. For these respondents, the saying “A picture is worth a thousand words” is particularly relevant considering the challenge of reading through the lengthy text descriptions to understand the vignette scenario (Hibbing & Rankin-Erickson, 2003).

In addition to ease of understanding, because respondents can process information shown in image vignettes relatively quickly, we expect that the use of image vignettes will reduce respondents’ cognitive burden and overall survey time. In turn, these two aspects could contribute to improving survey data quality by reducing survey break-offs and respondents’ satisficing behavior.

A second potential advantage of image vignettes is that they might help satisfy the measurement assumptions. For example, it has been found that first names used in text vignettes (e.g., “Alice falls asleep easily at night…”) can lead to respondents’ inferences about that person’s characteristics, such as age, gender and racial/ethnic information (e.g., Jürges & Winter, 2013). If respondents from different groups perceive the vignette person as having different characteristics, VE is likely to be violated. This may be of less concern in well-designed image vignettes where the physical characteristics of the vignette person are clearly presented, limiting the possibility of different interpretations. Note that the performances of image vignettes can largely depend on how they are designed. Some design features may be associated with different interpretations of the vignette person, e.g., respondents with different age and gender may view a vignette person with tattoos, piercings, and unnaturally colored hair differently. While it is true that not all image vignettes will help satisfy the measurement assumptions, in this study, we aim to investigate: with carefully designed image vignettes on health domains, whether image vignettes could help with measurement assumptions, compared to text vignettes.

Because there are no prior studies on the use of image anchoring vignettes, it remains an open question whether this approach can remedy limitations of current text vignettes. To fill this gap, this paper aims to evaluate the use of image AV as an alternative to text vignettes and to compare the performance of image and standard text vignettes in terms of response time, how well they satisfy the RC and VE assumptions, and their ability to reduce measurement errors in a confirmatory factor analysis (CFA) framework. In this paper, we focused on four health domains – sleep, affect, mobility, and pain – which are known to be subject to DIF (e.g., d’Uva, O’Donnell, & Van Doorslaer, 2008). We have three research questions (RQ).

RQ1: Will image AV reduce response time, compared to text AV? This research question will be addressed by analyzing survey time associated with text and image AV using time stamp data.

RQ2: Will image AV better meet AV measurement assumptions compared to text AV? This research question will be addressed by examining both VE and RC assumptions for text and image AV.

RQ3: In a confirmatory factor analysis (CFA) framework, a.) we will investigate whether a model of latent health based on image or text AV-adjusted scores will show better fit compared to a model based on unadjusted self-reported scores, and b.) whether a model based on image AV-adjusted scores will have similar or better fit compared to a model based on text AV-adjusted scores, i.e., will image AV adjustment achieve similar or better measurement error-reduction, compared to text AV?

## Methods

### Design of Image Vignettes

Prior to designing the image vignettes, we established criteria for image selection or creation. A three-step approach was used to develop these criteria: specifically, we 1) thoroughly examined critical elements of the four health domains, 2) identified common elements applicable across groups (e.g., arm pain) based on the literature review, and 3) based on the elements identified, we selected or designed images with these elements at different intensity levels for each domain (e.g., from no pain to extreme pain). Based on the developed criteria, images were then selected from commercial websites of images and photos (e.g., www.istockphoto.com/). In situations where, for a given health domain, no images meeting the criteria were found on those websites, we 1) recruited volunteers from different platforms (e.g., friends or family members) to serve as models in the photos, 2) obtained each volunteer’s consent to take a photo and to use it in this study, and 3) took the photo and edited them. To remove potential confounding effects of various image elements, such as background, size, resolution, and color balance, the selected images or photos were further edited by students with expertise in image-editing.

The ultimate goal of the image vignette design for the current study was to have three well-designed image vignettes per domain. For the purpose of selecting the most comparable images across cultures, we first designed six images for each characteristic: two images for each intensity level (e.g., two no/low pain, two moderate pain and two extreme pain vignettes) per design condition, and eventually selected three out of the six for each condition in the pretest. The selected images (see Appendix 1) were then used in the web survey experiment as described below^1^.

### Pretesting

The pretest was conducted through Amazon Mechanical Turk (MTurk), where we posted the survey announcement, also known as Amazon’s human intelligence tasks (HITs). Eligible respondents can browse the HITs and decide if they would like to take the survey or not. The announcement contains a link to the pretest survey, which was programmed with Qualtrics. The pretest was open to U.S. workers who were 18 or older. A $0.45 incentive was offered for each completed survey. To recruit respondents of all age groups, toward the end of the data collection, we posted a HIT open only to older respondents with the same incentive. In total, 201 respondents completed the pretest survey, about half of them aged 50 years or older. The main criteria applied to evaluate and select proper images was based on whether respondents could correctly rank order vignettes as expected. This method was first used by World Health Organization (WHO) in their pretesting of anchoring vignettes (Murray et al., 2003). For the two sets of image options, the image with the higher correct ranking rate (the percentage of respondents who correctly ranked the vignette series) was selected. The final correct ranking rates ranged from about 80% to 97% across all health domains.

### Web Survey Procedure

The main data collection was based on a web survey using a non-probability online panel. Respondents from four different racial/ethnic groups – Non-Hispanic (NH) white, NH black, English-speaking Hispanic and Spanish-speaking Hispanic – were recruited through Qualtrics’ online survey panel, which partners with over 20 Web-based panel providers to supply diverse, quality respondents (more information about Qualtrics survey panel, see also Holt & Loraas, 2019; Ibarra et al., 2018). The reason for including these groups is that race/ethnicity and language are proxies of cultures (Davis et al., 2019; Lee et al., 2014; Lee et al., 2017) and are known to influence respondents’ self-reporting of their health status (McCarthy, Ruiz, Gale, Karam, & Moore, 2004; Lee et al., 2014). For example, Hispanics have been shown to conceptualize health differently than non-Hispanic Whites as they “include non-medical aspects, such as spiritual and social wellbeing, in addition to medical conditions that non-Hispanic Whites consider the most critical element for assessing health” (Lee et al., 2014). Language can also influence respondents’ reporting of their health status, e.g., Lee and colleagues examined Hispanics’ self-reported health by interview language and found that the difference was primarily due to Hispanics interviewed in Spanish (Lee et al., 2014). Respondents from each racial/ethnic group were randomized into three conditions: the standard text vignette condition and two image vignette conditions that differed in the vignette persons’ characteristics (See Appendix 2 for a flowchart of the experimental conditions and assignments). Robustness of randomization was examined, and results show that there are no significant socio-demographic differences across the experimental conditions (Supplemental Table 5), suggesting that the randomization works well.

For the text vignette condition, we adapted the text vignette descriptions from those widely used in many major surveys (e.g., HRS). Each domain had a series of three vignettes, describing different intensity levels of the measured construct: low, moderate and high (e.g., from least to most pain). For the image condition, we used the image vignettes designed and selected in the pretest with three vignettes per condition, depicting three levels of difficulty/intensity of symptoms in each domain (see Appendix 1). The introduction to the vignette questions also followed the standard approach used in earlier surveys such as HRS. We randomized the order of the domains and of the three vignettes per domain presented to respondents in order to isolate question order effects. Besides self-assessment and vignette questions, the study also included responses to objective questions regarding these health domains, time stamp data, and respondents’ demographic and socio-economic information.

In translating the instrument into Spanish for Spanish-speaking Hispanics, this study followed the set of best practices developed by the United States Census Bureau (Pan & De La Puente, 2005) and the Cross-Cultural Survey Guidelines developed by the survey research center at the University of Michigan (Mohler et al., 2016). Translation was conducted by the translation team of HRS. The translated questionnaire was then reviewed and tested by 20 bilingual speakers who are native Spanish speakers and are also fluent in English.

The online survey questionnaire was programmed in Qualtrics. The Qualtrics online panel team sampled respondents from their panel. Except for Hispanics speaking Spanish, around 750 respondents were sampled for each of the three other race/ethnic groups. Each of the three sampled subgroups had nearly equal proportions of 1) male and female, 2) below or equal to high school education and higher than high school education, and 3) respondents aged 18–49 or 50 and over. For Spanish-speaking Hispanics^2^, 889 respondents were sampled with about 43% male respondents. Detailed information of the sample profile is presented in Table 1. In conducting this experiment, we implicitly make the stable unit treatment value assumption (SUTVA) that the outcome for one respondent is unaffected by the assignment of treatments to the other units. This assumption is likely to have been met in our study given Qualtrics’ large pool of respondents and our duplicate check on respondents’ IP addresses.

Email invitations were sent to selected respondents, with the link to the survey included in the email. Respondents from each racial/ethnic group were randomly assigned to one of the three vignette type conditions, one text condition and two image conditions.

### Analysis Strategy

We first examined the distributions of the self-assessment and vignette questions by vignette type for each domain descriptively. We then examined whether and to what extent the self-assessments were affected by DIF following previous literature studying measurement errors in self-assessed health (Yan & Hu, 2018). Specifically, since self-assessments of health are correlated with objective health conditions (Idler & Kasl, 1995), we take advantage of this relation to gain insights on how DIF affects respondents’ uses of the scales. We constructed a measure of objective health for each domain using respondents’ own answers to a series of factual questions asking about health conditions for each domain. We then standardized the number of health issues (e.g., the number of mobility issues) within each racial / ethnic group. The resultant standardized score reflects the number of standard deviations above or below the racial/ethnic subgroup mean, where a value of 0 stands for the subgroup average. Negative values of health scores denote better health than the subgroup average (i.e., respondents reported fewer health conditions) whereas positive values indicate worse health than the racial/ethnic subgroup average (i.e., respondents reported more health conditions). For each category selected on the self-assessment question, we computed the mean of the standardized scores and compared them across different racial / ethnic groups.

We then examined RQ1 to RQ3 as described below. Note that in examining RQ1 to RQ3, the variables were not standardized.

#### RQ 1.

To evaluate whether image vignettes can reduce survey time compared to text vignettes, we analyzed the survey time using time stamp data. The mean response time was compared between the text and image vignette types. To formally test the effects of vignette types on survey time, for each domain, we fit multilevel linear regression models with random intercepts. The log-transformed response time was used as the outcome, given that time is right skewed. In this model, Level 1 corresponds to vignette questions, and Level 2 corresponds to respondents. Level 1 covariate was vignette type (image vs. text vignettes) and Level 2 covariates included respondents’ demographic and socio-economic variables. Results of the multilevel model can be found in Appendix 3 (Supplemental Table 6). Given that it is hard to ascertain whether respondents were completing the online survey from beginning to the end in one sitting or took temporarily breaks – e.g., checking emails and browsing other web tabs, we employed a two-step procedure to identify response time outliers. First, based on the response time distribution, we used 15 minutes (i.e., 900 seconds)^3^ per vignette question as a threshold to identify those who might took a break during the survey completion. Second, we examined distributions of random effects and residuals of the multilevel models described above. Using histograms and Q-Q plots, outliers on these parameters were inspected visually. In total, the first step identified four response time outliers for pain domain, two outliers each for sleep and mobility domains and six outliers for affect domain were identified and excluded from this analysis. The second step did not identify any outliers.

#### RQ 2.

We compared image and text vignettes in terms of how well they satisfy the two measurement assumptions – VE and RC. Below we describe approaches for each of the two assumption-testing.

#### RQ 2a (Test for VE).

Two tests of VE were conducted. The first one is referred as correct rank ordering test, which examines whether respondents could correctly rank order vignettes based on their intensity level. Several previous studies refer to this test as a weak test for VE, stating that correct rank-ordering is a “necessary but not sufficient” condition for VE (e.g., Grol-Prokopczyk et al., 2015; Kristensen & Johansson, 2008), given that if VE is fulfilled through effective vignette design, respondents should agree on the ranking of the vignettes.

It is possible that respondents may rate two or three vignettes identically. For example, if a respondent has a very high threshold for what is “mild” pain, that respondent may rate the first two vignettes (low and moderate pain) or all vignettes as no pain. This is referred to as “ties” in vignette-ratings. Although it is possible that a respondent may have *true* ties for all three vignettes (i.e., view the three vignettes as having similar intensity levels and rate them identically), this is unlikely given the differences among the intensity levels in the vignette design. Thus, here we only consider two kinds of ties: 1) ties between the first two vignettes (low and moderate intensity) and 2) ties between the last two vignettes (moderate and high intensity).

The second test for VE was a statistical test conducted following Grol-Prokopczyk (2018). This method was first developed by d’Uva et al. (2011) and applied in many other studies (Grol-Prokopczyk, 2018; Grol-Prokopczyk et al., 2015; Molina, 2016). The rationale behind this test is that if respondents view each vignette in the same way (VE), the distance between any two vignettes on the latent dimension should be the same for all respondents (d’Uva, Lindeboom, O’Donnell, & van Doorslaer, 2011). The test is based on a likelihood-ratio (LR) test of two nested models. Both models are variations of the hierarchical ordered probit (HOPIT) model. Below we list the key differences between the two models. The first model, Model (A)^4^, predicts a respondent’s perceived location of vignettes:

(A) $${V_{ij}}^{*}=\alpha_{j}+\varepsilon_{ij}$$

where ${V_{ij}}^{*}$ is respondent *i*’s perceived location of vignette *j* on the latent dimension, *α*_*j*_ is a constant term and *ε*_*ij*_ is the random error term that is assumed to be normally distributed with mean zero and variance one. For one of the vignettes in a domain (the reference vignette), *α* is set to 0 for model identification. The cut points (*τ*) for the vignettes are modeled in the same way as in the HOPIT model. Note that Model A does not include covariates to predict perceived vignette locations on the latent dimension. This is consistent with VE, namely that respondents’ perceptions of vignettes do not depend on their background and are constant across different population groups.

In the less restrictive Model B, a vector of covariates, ***X***_***i***_, is added to Model A to predict the perceived vignette locations. In this study, ***X***_***i***_ includes marital status, employment status, age, gender, education, income level, and racial/ethnic group.

(B) $${V_{ij}}^{*}=\alpha_{j}+\lambda_{j}X_{i}+\varepsilon_{ij}$$

Since this model is not identified, one needs a normalization. For one of the vignettes (the reference vignette), both α and ***λ***_***j***_ are set to zero for identification. If VE is satisfied, ***λ***_***j***_ will be 0 for each *j*. Model A is nested in Model B and if VE is satisfied, the LR test will not reject Model A. If, however, the LR test rejects Model A, it indicates that respondents with different characteristics perceive the severity of the vignettes differently. The estimated coefficient vector ***λ***_***j***_ will indicate which covariates are driving the violation of VE.

#### RQ 2b (Test of RC).

Our test of RC was conducted following Grol-Prokopczyk et al. (2015). This test was based on visual comparisons of two sets of predicted cut points. One set was generated from vignettes only, based on Model A as in the tests of VE. The other set was generated from self-assessments based on Model C below, which uses objective health measures to predict the self-assessments.

(C) $$Y_{i}^{*}=\mu +\beta W_{i}+\varepsilon_{i}$$

where $Y_{i}^{*}$ is respondent *i*’s true score on the latent dimension in the measured domain, *μ* is a constant term and *ε*_*i*_ is a random error term that is assumed to be normally distributed with mean zero and variance one. ***W***_***i***_ is a vector of covariates consisting of the objective measures. The cut points are modeled in the same way as in Model A. The predicted mean cut points from the two models were then graphed in a figure for visual comparisons. The RC test basically compares the shape (Grol-Prokopczyk et al., 2015) of the two sets of cut points. A similar shape would indicate that respondents had similar standards when rating vignettes and rating themselves (RC). As mentioned in Grol-Prokopczyk (2018), this test can be viewed only as suggestive. The objective measures used in this study include: whether respondents have seen a doctor about their difficulties with sleep, whether respondents on average sleep less than 7 hours or over 9 hours each day, a sleep quality score^5^, total pain index^6^, number of mobility activities that respondents have difficulty with, number of chronic health conditions, and the Kessler Psychological Distress Scale (K6) (Kessler et al., 2002).

#### RQ 3.

The self-assessments for the health domains have often been used in a confirmatory factor analysis (CFA) framework to measure latent overall health. To examine whether AV-adjustment can reduce measurement errors in self-assessments, following Weiss & Roberts (2018), we compared the model fit of the CFA using original responses with the CFA using text / image AV-adjusted scores. If the use of AV-adjusted scores can correct DIF, we would expect the models with AV-adjusted scores to have better fit (RQ 3a; see also Weiss & Roberts, 2018). To evaluate whether image AV can achieve similar or better DIF-correction compared to text AV (RQ 3b), we also compared the magnitude of improvement compared to CFA with original self-reports, for both image and text AV-adjustment.

The AV-adjusted scores were calculated using the non-parametric approach, following previous literature (Wand et al., 2011). In situations where respondents have ties in their AV-rating or inconsistent AV orders from researchers’ expected order (i.e., order violations), the non-parametric method will result in an interval instead of a number for these respondents. Following the recommendations in previous literature (Kyllonen & Bertling, 2014; Primi, Zanon, Santos, De Fruyt, & John, 2016; Weiss & Roberts, 2018), the lower bounds of the intervals are chosen as the adjusted scores for respondents with ties or order violations. Model fit criteria including Comparative-Fit-Index (CFI), Tucker–Lewis index (TLI), and a Root Mean Square Error of Approximation (RMSEA) and 90% confidence interval (CI) of RMSEA are used to compare the models (Schreiber et al., 2006). A CFI greater than 0.95 and a TLI greater than 0.95 are considered as acceptable model fit (Hu & Bentler, 1999). A RMSEA less than or equal to 0.05 is considered as good fit, and less than or equal to 0.08 is considered as moderate fit (MacCallum, Browne & Sugawara, 1996). For the 90% CI of RMSEA, ideally the lower value should be less than 0.05 and the upper value less than 0.08 (MacCallum, Browne & Sugawara, 1996; Schreiber et al., 2006).

## Results

### Descriptive Analysis

**Figure 2:**
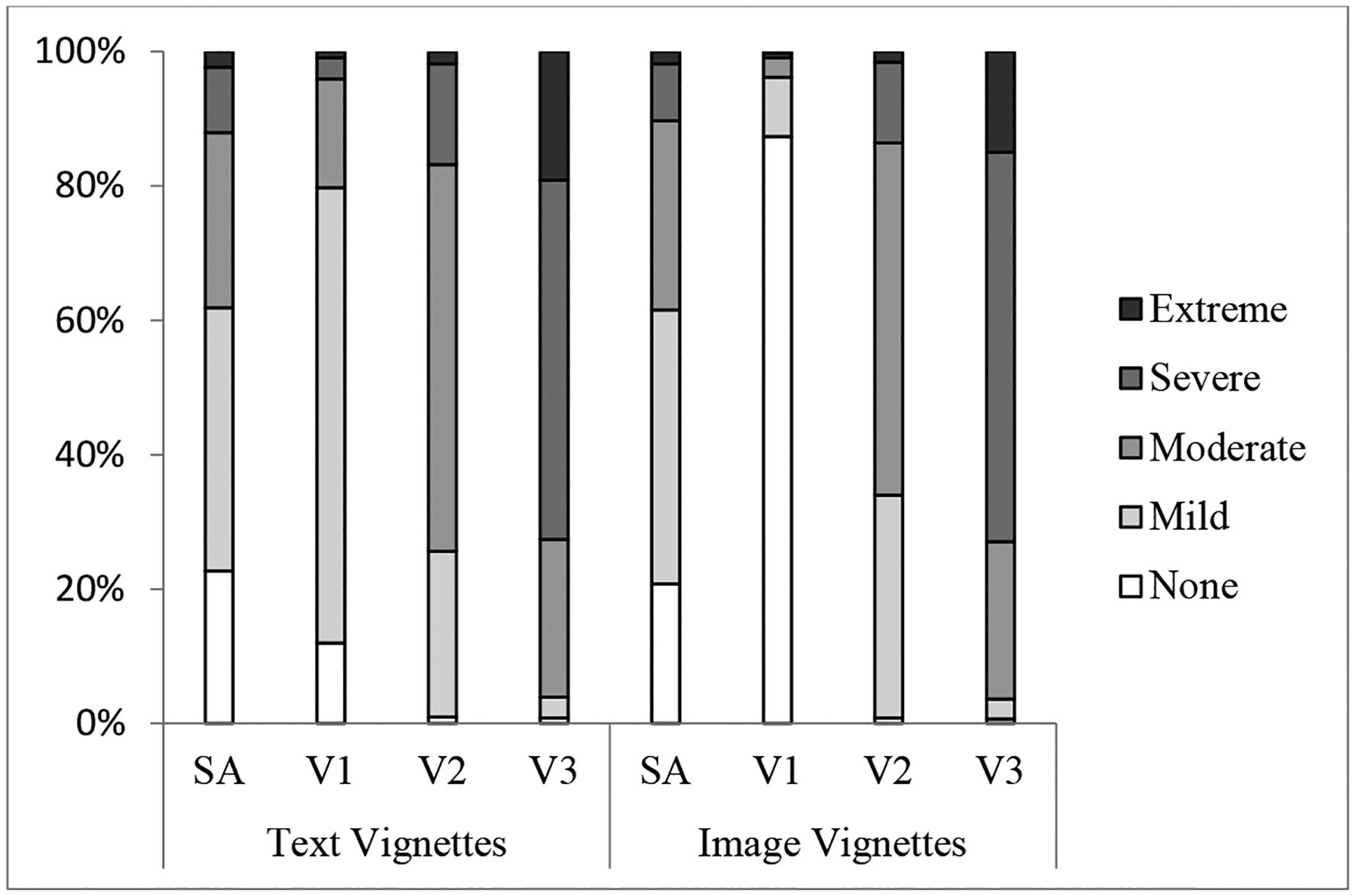
Responses to pain self-assessment (SA) and difficulty/intensity questions for three vignettes (V1 = none/mild; V2 = moderate; V3 = severe/extreme).

We first examined the distributions of the self-assessment and vignette questions by vignette type for each domain. Figure 2 shows the distribution for the pain domain. Similar patterns were found for other domains. As expected for a properly randomized design, for each domain, the distributions for the self-assessment questions do not differ by vignette type-text or image vignettes. Comparing vignette distributions by vignette type, in general, the intensity levels of the image vignettes can be better differentiated than those of the text vignettes.

### DIF Evaluation

**Figure 3:**
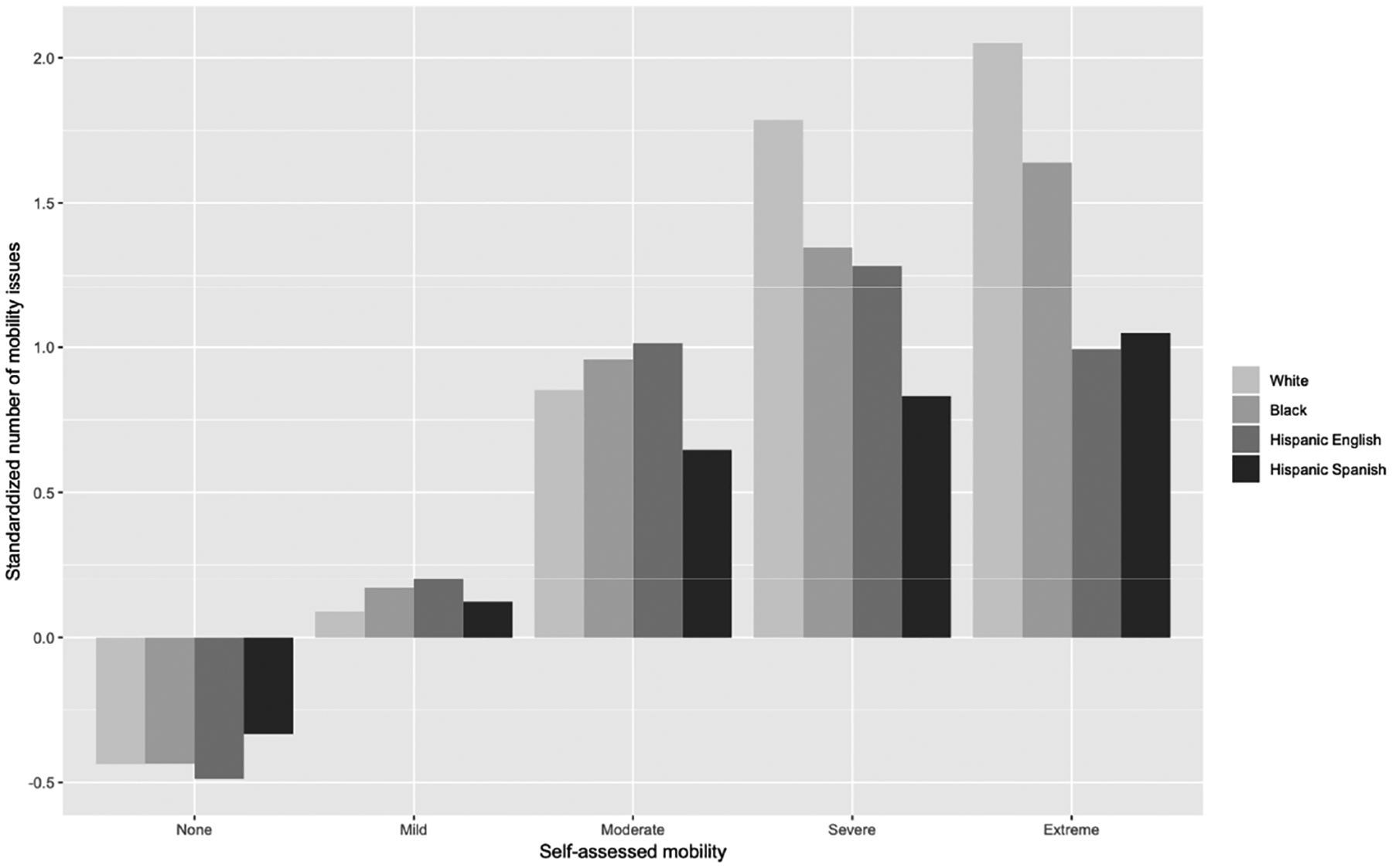
Mean standardized number of mobility issues by reported response categories of self-assessed mobility.

We then examined whether DIF was present in the self-assessments^7^. Figure 3 displays the mean standardized number of mobility issues by reported response categories of self-assessed mobility. For all four racial / ethnic groups, the mean standardized scores are negative for those who selected “none” for mobility, and positive for those who selected “mild” or “extreme” mobility issues. For White respondents, the biggest increase of the mean standardized score occurs between “Moderate” and “Severe”, while the change of the score from “Severe” to “Extreme” is much smaller. Compared to White respondents, for Black and Hispanic speaking Spanish, the change of the mean scores from “Moderate” to “Severe” is similar to change from “Severe” to “Extreme”. Note that for Hispanics speaking English, the mean score is lower among those who select “Extreme” compared to those who select “Moderate” or “Severe”, while for all other groups, the standardized score increases as the severity of the response categories increase. This indicates that respondents from different racial / ethnic groups use the scales differently, leading to DIF, and indicates the need to use methods like anchoring vignettes to achieve cross-cultural comparability.

#### RQ 1. Response time

As shown in Table 2, regardless of domain, the average time respondents spent on a text vignette question is about twice as long as time spent on an image vignette question. Results for the statistical test of differential response time by vignette types using multilevel models are presented in Appendix 3 (Supplemental Table 6), which show consistent results as Table 2.

#### RQ 2a. VE Test

Results of two tests of VE, the correct rank ordering test and the VE statistical test, were presented below.

#### Correct Rank-Ordering.

Table 3 shows the percent of respondents whose ratings for the vignettes are consistent with the expected order (i.e., low intensity to high intensity). The percentages ranged from 17% to around 82%, depending on the domain. It is noted that for each of the four domains, the percentage of consistent rankings is significantly higher for the image than for the text vignette condition. In other words, respondents assigned to the image conditions are more likely to agree on the rank order of the vignettes than those assigned to the text condition. Respondents seem to have difficulty differentiating the rank orders of sleep and mobility *text vignette*s, with less than 20% able to correctly rank vignettes for these domains^8^. We also formally tested the effects of vignette types on the rank ordering of vignettes by fitting logistic regression models for each health domain (Results not shown). Not surprisingly, the odds of correctly ranking vignettes in the image vignette conditions are significantly higher compared to those in text vignette conditions. This is consistent across all four domains. Similar results were found when allowing for ties.

#### Statistical test of VE.

Table 4 presents the results of statistical test of VE. The VE assumption is rejected in almost all conditions, except for the sleep text vignettes.

Table 5 presents the results for predicting vignette locations (i.e., where it lies on the latent health spectrum) for both text and image vignette conditions of each domain^9^. In Table 5, Vignette 3 is the reference vignette, the one describing the highest pain level. Gender, marital status and racial/ethnic groups are the main predictors that drive the violations of VE for pain text vignettes. As for pain image vignettes, gender, age, income, and racial/ethnic groups are the main predictors that drive the violations of VE.

Those who are married view the first pain text vignette (the vignette with the least pain) as further away from the reference vignette on the latent spectrum, with a positive coefficient of 0.31 (*p* = 0.02). In other words, married respondents view the first pain text vignette as depicting better health (or less pain) than those who are not married. Males view the first pain text vignette as depicting worse health (or more pain) than females, which is consistent for both text and image AV conditions. Note that racial/ethnic group differences are significant for all health domains, suggesting that respondents from different racial/ethnic groups view the vignettes differently. For example, Hispanics interviewed in Spanish view Vignette 1 as depicting more pain than White respondents, regardless of text or image vignette designs.

**Figure 4:**
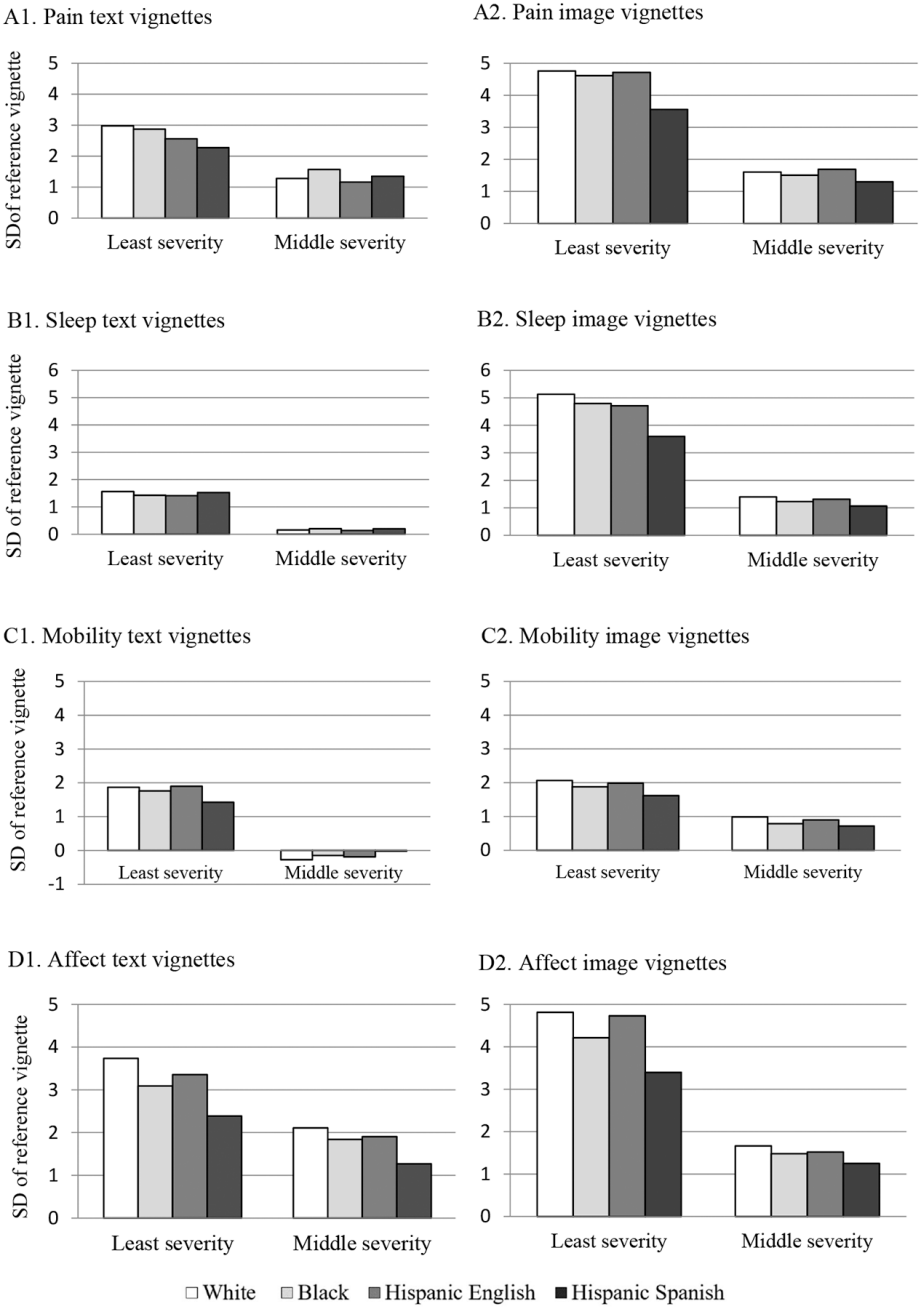
Estimated vignette locations, compared to the reference vignette (severity 3) on the latent health spectrum (measured in standard deviations of the reference vignette) for each health domain. Zero on the y-axis represents the mean of the reference (most pain or least healthy) vignette; higher numbers represent better perceived health.

As shown in Table 5, racial/ethnic group is a predictor that drives violations of VE for all health domains. To further examine this, Figure 4 presents the estimated vignette locations relative to the reference vignette by racial/ethnic group and vignette type for each health domain. If VE is satisfied, we would expect the estimated pain vignette locations to be exactly the same for each racial/ethnic group. This is not the case, as can be seen from Table 5 and Figure 4. As shown in Figures 4A1 and 4A2, Hispanics who completed the Spanish-language survey view the first vignette person (least severity) as having more pain (i.e., closer to 0 line, the reference vignette with the highest severity) compared to White respondents. On the other hand, Hispanics who completed the English-language survey also view the first vignette person as having more pain than do White respondents under the text condition, but not under the image vignette condition. Similar results are found for the affect domain (see Figure 4D1 and 4D2).

Figures 4B1 and 4B2 shows the estimated vignette locations for the sleep domain. As can be seen from Figure 4B1, the estimated vignette locations across racial/ethnic groups are very similar, indicating that respondents regardless of racial/ethnic background view the vignettes in similar ways. However, it is worth noting that the perceived vignette location for the second vignette is not significantly different from the reference vignette, suggesting that the sleep text vignettes failed to provide a good distinction between the second and third vignettes. As shown in Figure 4B2, despite the VE violation (e.g., Hispanics who took the Spanish survey view the first vignette person as having more sleep difficulties compared with White respondents), image vignettes did a much better job differentiating the intensity levels of the three vignettes. Similar results are found for mobility domain (see Figures 4C1 and 4C2).

#### RQ 2b. RC-Test

**Figure 5:**
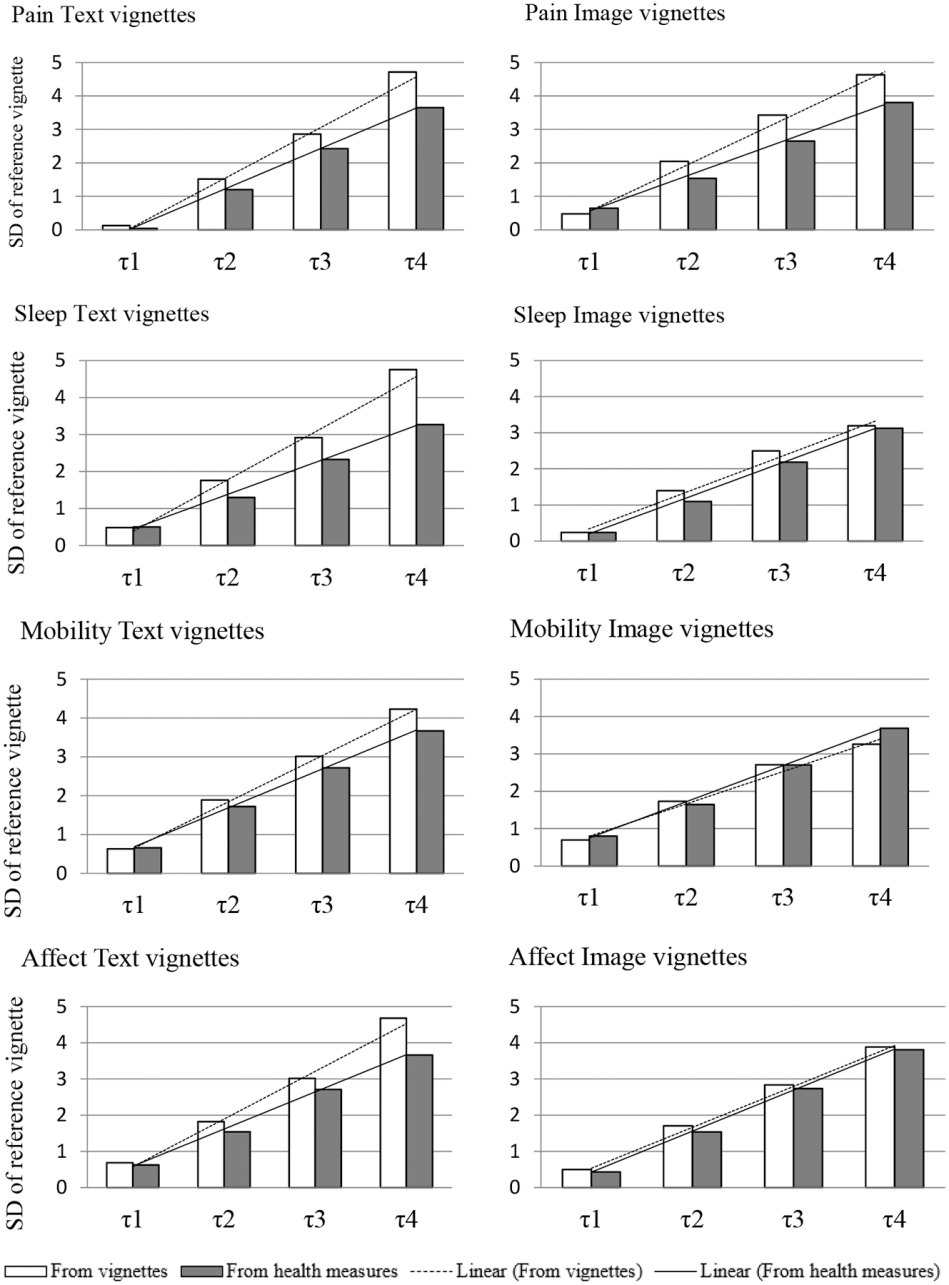
Estimated cut points for health domains based on vignettes and health measures. Evaluations are based on comparisons to the reference vignette [highest severity; measured in standard deviations (SD) of the reference vignette]. τ1–τ4 are cut points for the five-point response scale from “None” to “Extreme” (e.g., τ1 is the cut point between “None” and “Mild”).

As described in the Analysis section, the RC assumption test is based on visual comparisons of two sets of predicted mean cut points: one from Model A which has only vignettes (i.e., no self-assessments included in the model) and another from Model C which includes self-assessments and objective measures. Figure 5 shows the estimated cut points for all four health domains. If the vignette-derived cut point patterns are similar to the health measures-derived cut points, this indicates no or only minor violations of RC. For pain domain, both text and image vignettes show minor violations of RC. For all other three domains, image vignette conditions seem better fulfill RC, compared to text conditions.

#### RQ 3. Confirmatory factor analysis before and after anchoring vignette-adjustments

To test whether image and text AV-adjusted scores perform better than original scores (RQ 2a.), we compared model fit indices in a confirmatory factor analysis (CFA) using both adjusted scores and original scores. The cutoff criteria for acceptable fit are presented in the Analysis Strategy section. As shown in Table 6, CFI are above 0.95 and TLI are around or above 0.95 for all models, indicating that the models fit the data well for all the conditions. Models with AV-adjusted scores lead to better (i.e., higher) CFI and TLI values. For example, for the image condition subsample, the TLI of the model with image AV-adjusted self-assessment scores is 0.977, which is higher than the TLI of the model with original self-assessments – 0.942. RMSEA results shows that using both text and image AV-adjusted scores can greatly improve RMSEA. This suggests that using both text and image-adjusted scores improve CFA model fit.

To test whether image AV-adjusted scores perform similar or better than text AV-adjusted scores in the CFA framework (RQ 2b.), we assessed the model fit indices in CFA with text AV-adjusted scores and CFA with image AV-adjusted scores. As shown in Table 6, both text and image AV-adjustment improves the CFA based on original self-reports with similar improvements in terms of model fit indices. In addition, the CFI, TLI and RMSEA results are similar across the two CFA models with text vs. image AV-adjusted scores. The CFA with image AV-adjusted scores have better 90% CI of RMSEA (which ideally should have the lower value less than 0.05 and the upper value less than 0.08).

## Discussion

This study examines the use of image anchoring vignettes (AV) to adjust DIF in self-assessments of health. Despite the fact that text AV have been adopted in many comparative studies, there are several critical challenges associated with text AV. To explore ways to overcome these challenges, this paper proposes the use of image AV, consisting of carefully designed and pre-tested images. In this study, the performances of text and image AV are compared with respect to a number of properties, including response time, tests of assumptions, and CFA model fits. Overall, the results suggest that the image AV methodology can be used as an improved and effective alternative to text AV in cross-cultural research, although the extent to which the VE assumption is satisfied needs further investigation for both text and image AV.

Specifically, the use of image AV can reduce survey time to about half the time of text AV. This result is consistent with previous literature on differences of information processing between text vs. image stimuli (Azizian et al., 2006; Naspetti et al. 2016; Schlochtermeier et al. 2013). Survey time is an important indicator for respondent cognitive burden, which can influence survey data quality and survey response rates. Survey time is also closely associated with survey cost, with shorter time potentially implying lower survey costs. Thus, image AV offers a time and potentially cost-efficient survey option, compared to text AV, especially in studies with many AV items (e.g., Weiss & Roberts, 2018).

Results for comparing how well AV assumptions are satisfied between text and image AV show mixed findings. On the one hand, image AV outperforms text AV in that respondents can better distinguish the different intensity levels in image vignettes (e.g., from no pain to extreme pain) than in text vignettes, indicating that respondents are more likely to perceive the vignettes in similar ways and in the designed order in the image AV condition compared to the text AV condition. This finding is consistent with previous literature showing the information processing advantage of emotional images in terms of larger or more pronounced emotion effects evoked by image stimuli, compared to text stimuli (e.g., Schlochtermeier et al., 2013). One of the reasons may be that image vignettes lead to a stronger activation of relevant information in the cognitive system resulting in more arousal and perceived intensity. Another possible reason is that text AV puts a higher cognitive burden on respondents, potentially resulting in more satisficing behavior including straight-lining (i.e., respondents select the same response option for all the vignette questions) and random selection of responses. For example, we find that respondents assigned to the text vignettes treatment are more likely to straight-line than those assigned to image vignettes (results not shown).

On the other hand, for both text and image AV, it is found that respondents’ perceptions of the vignettes can differ by cultural subgroups, a violation of VE. Similar to text AV, various factors may cause violations of VE for image vignettes. First, like text vignettes, the information in image AV may serve as memory cues that can trigger other related memories, leading to differences in perceptions. Second, although elements included in image AV may be more easily standardized than text AV (e.g., gender of the hypothetical person), the included elements may still weigh differently for different subgroups. For example, an element in the image may be more familiar to one cultural group than to another, resulting in perception differences. The violation of VE implies that designing “universal” anchoring vignettes (Grol-Prokopczyk, 2018), which are familiar to all population groups and reveal the same information to all respondents, is still a challenge for both text and image vignettes.

Despite the VE violations, results of the CFA models indicate that, compared to the model with self-reported data, using vignettes-adjusted scores can greatly improve model fit, which is consistent with Weiss & Roberts (2018)^10^. This shows that, even though VE is not met, it is still better to use text or image AV-adjustments, which can effectively reduce measurement errors. Comparing the two vignette types, text and image vignettes perform similarly in terms of measurement error reduction in the CFA models.

Given the clear advantage of image vignettes in reducing survey time, lowering respondents’ cognitive burden and better differentiating intensity levels, we believe there is a potential for the use of image AV to improve text AV methodology.

This study also revealed important findings to deepen our understanding of the vignette methodology, including how different respondents view and rate vignettes. For example, it was found that male respondents view the first pain vignette as describing more pain than female respondents do (as shown in Table 5). This may be because females experience more pain than males (Cepeda & Carr, 2003). They may use themselves as a standard of comparison when rating the vignette person and thus view the first vignette person as depicting minimal pain. Due to space restrictions, this study will not discuss detailed results for all covariates. Future studies can look into this further. In addition, this study generates new insights into the design and use of image AV, and the designed image AV items can be applied to other studies that use anchoring vignettes to adjust self-reported health.

It is worth mentioning that this study is limited in several ways. First, due to resource constrains, our experimental study is based on a non-probability sample, from which the results were not intended to generalize to the full U.S. population. Among the four types of validity of causal inference (statistical, internal, external and construct validity) in Shadish, Cook and Campbell (2002), this paper focused on the internal and statistical validity with a randomized experiment to compare DIF-adjustment results between vignette types. Per Edgington (1966) and Berk et al. (1995), randomized experiments permit statistical inferences about the experimental factors. However, due to the nature of the sample, we do not claim that our results generalize to the complete U.S. population and beyond. Future studies could replicate this study in probability-based representative surveys to evaluate the effect sizes of the group comparisons in the population. Second, the current RC test is not based on a statistical test and additional evaluations of RC using more stringent RC test are needed (Grol-Prokopczyk, 2018). Third, the objective health measures used in the RC tests may not fully capture actual health. One may also argue that these objective health questions are based on self-reports and may be subject to reporting errors. Note that the questions about objective health are straightforward factual questions (e.g., whether respondent has received doctor diagnosis of certain diseases), for which reporting errors may be less of an issue compared to self-assessing of a health domain. Also, many of the objective measures used in this study are based on widely-used existing scales, and have been successfully applied in previous literature (Kessler et al., 2002; Ray et al., 2009). If available, future studies could use bio-markers (e.g., medical test results and genetic data) in the RC tests. Fourth, this study examined the most commonly used text vignettes that are included in HRS, SHARE, and many other large-scale surveys. It is possible that text vignettes with differently-worded descriptions may perform better in tests of assumptions than the current text vignettes. The same may be true for image vignettes. Possibly, better-designed pictures are less likely to lead to rejection of the VE and RC assumptions. Future research could compare text and image vignettes with different descriptions or designs.

Our research suggests several important directions for future research. First, this study focuses on the comparisons of text and image vignettes in correcting for DIF. Future research could examine in detail how different image vignette designs may influence the performance of image AV. For example, in a related study, we found that when rating image vignettes with average body size vs. obese for the mobility domain, respondents tend to rate the obese vignette person as having more mobility difficulties than a vignette person with average body size. This is not surprising given that obese individuals are more likely to have mobility limitations than non-obese individuals (Koster et al., 2007). In addition, the vignette images showing average body sizes, which match the body size of the majority of respondents, show a higher rate of consistency in the rank-orderings, indicating that respondents may better perceive the image vignettes when the vignette figures match more closely their own characteristics. This could shed light on the future design of image vignettes. For example, it indicates that image vignettes that have a broader applicability and familiarity to the respondents may better satisfy the assumptions. Future research could further evaluate the effects of a wide range of vignette characteristics on image vignette performance. Second, given budget constraints, all respondents in this study are from the U.S. Future research could evaluate the use of vignettes in a less homogeneous group, such as extending the study to cross-national surveys and/or to a wide variety of other racial/ethnic groups, such as Asians, American Indian or Alaska Native, and Native Hawaiian or Other Pacific Islander. Third, some domains may be too complex to be expressed using images, such as self-reported political attitudes. In addition, using static image vignettes may not be the best way to present measures related to change over time and location, such as a slow or fast walking speed. Future research can evaluate other visual vignette designs such as using short videos in web surveys (Banuri et al., 2018; Mendelson, Gibson, & Romano-Bergstrom, 2017) and the use of visual vignettes in different domains, including domains that cannot be easily visualized using static images. Fourth, the ways vignettes are presented and their applications can vary by survey mode, which may influence their performance. Verbal vignettes can be delivered orally in telephone and face-to-face interviews or visually as text in mail and web surveys, but image vignettes have to be presented visually in mail and web surveys, or as a picture presented by interviewers in face-to-face surveys. Future research could evaluate mode effects for both text and image vignettes.

In conclusion, this study indicates that using either text or image AV adjustments can reduce measurement errors compared to the analysis without using any AV, and the use of image AV can greatly reduce survey time and respondents’ cognitive burden as compared to text vignettes. Improving VE, (in other words, minimizing different interpretations of vignettes by different groups), is critical for both text and image AV and requires further investigation. This study has advanced knowledge of the design and applications of image AV in health surveys and has implications for designing image AV of other domains. Future implementations of AV can use the findings of this study to introduce efficiencies in their survey designs.

## Supplementary Material

1

## Figures and Tables

**Figure 1:**
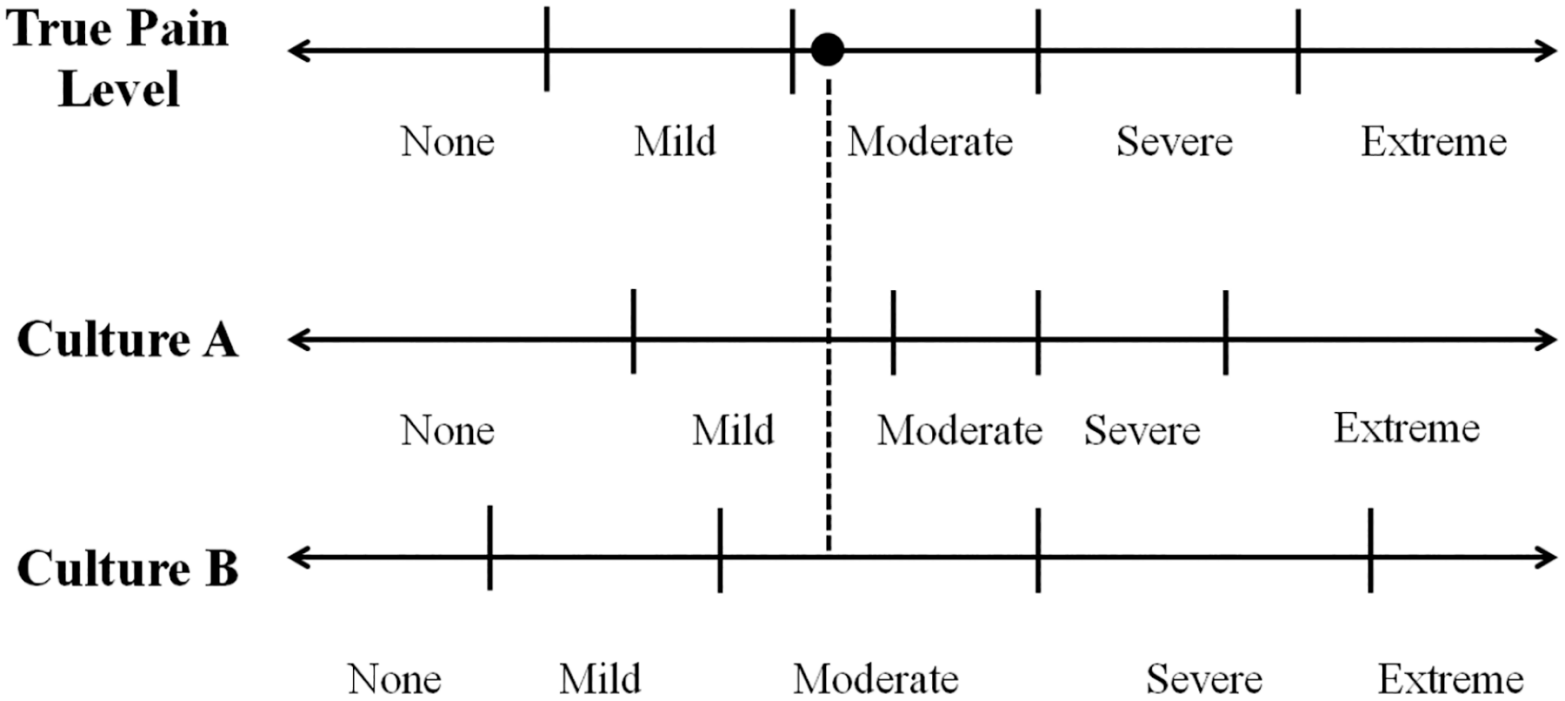
DIF for cross-cultural studies. Adapted from Hu, Lee & Xu (2018). The horizontal lines with arrows indicate the continuous scales of the domain (pain level). The short vertical lines indicate the cut points respondents used to answer the self-assessment question. The vertical dashed line indicates respondents responses to self-assessment questions. If a respondent’s pain level falls on that line, it indicates that they have the same true pain level.

**Table 1:** Respondents’ characteristics.

	White	Black	Hispanic-English	Hispanic-Spanish
	(n=760)	(n=750)	(n=750)	(n=889)
	%	%	%	%
Male	50.39	50.00	50.00	42.52
Age				
Age 18 – 29	14.34	22.80	22.13	21.37
Age 30 – 49	33.68	25.73	26.53	35.77
Age 50 – 64	30.13	36.27	34.53	33.52
Age 65 and above	21.84	15.20	16.80	9.34
More than high school	49.47	50.00	50.00	57.82
Married	53.42	36.67	50.93	54.78
Employed	50.92	52.00	56.13	57.14
Income				
Income below $40,000	35.00	35.87	33.07	34.76
Income between $40,000 - $69,999	33.95	42.93	41.33	45.67
Income $70,000 or more	31.05	21.20	25.60	19.57

**Table 2:** Average time (in seconds) spent on one text or image vignette question by health domains.

	Pain		Sleep		Mobility		Affect	
	Mean	SD	Mean	SD	Mean	SD	Mean	SD
Text vignette	15.93	8.81	15.73	8.25	17.85	10.31	18.05	10.59
Image vignette	7.95	3.58	8.38	3.61	8.33	3.79	7.42	3.17

**Table 3:** Percentage of respondents ordering vignettes consistently with expected ordering.

	Pain		Sleep		Mobility		Affect	
	n	%	n	%	n	%	n	%
Text vignette	1051	47.6	1051	17.7	1051	19.8	1051	67.1
Image vignette	2098	79.7	2098	74.0	2098	43.4	2098	81.8

**Table 4:** Likelihood ratio tests of vignette equivalence.

	Pain		Sleep		Mobility		Affect	
	df	LR Test	df	LR Test	df	LR Test	df	LR Test
Text vignettes	24	70.4***	24	24.4	24	55.1***	24	110.9***
Image vignette	24	137.4***	24	158.8***	24	67.1***	24	154.3***

*: *p* < 0.05;

**: *p* < 0.01;

***: *p* < 0.001.

**Table 5:** Predictors for perceived vignette locations on the latent health spectrum.

	Pain		Sleep		Mobility		Affect	
	Text	Image	Text	Image	Text	Image	Text	Image
*Vignette 1 (no/mild difficulty/intensity)*								
Constant	3.20***	5.12***	1.48***	5.51***	2.03***	2.04***	4.01***	5.72***
Married	0.31*	0.24	0.02	−0.13	0.27	0.06	0.37**	0.23
Male	−0.49***	−0.36**	−0.29**	−0.15	−0.30**	−0.05	−0.58***	−0.23
Employed	−0.1	0.00	0.06	0.20	−0.05	0.18*	0.06	0.15
More than high school	−0.09	0.09	0.16	0.22	0.02	0.18**	0.10	−0.24
Age 18 – 29	0.25	−0.57*	0.14	−0.53*	−0.08	−0.08	−0.05	−0.90***
Age 30 – 49	−0.17	0.01	0.14	−0.10	−0.10	−0.06	0.07	−0.69**
Age 50 – 64	0.09	−0.50*	0.15	−0.29	0.08	−0.10	0.05	−0.88***
Middle income	0.04	−0.28*	0.05	−0.43**	0.03	−0.02	−0.24	−0.33*
High income	−0.15	−0.15	−0.02	−0.29	−0.34*	−0.32**	−0.61**	−0.41*
Black	−0.12	0.02	−0.17	−0.25	−0.13	−0.19	−0.65**	−0.51**
Hispanic (English)	−0.47**	0.04	−0.21	−0.38	−0.04	−0.09	−0.46*	0.00
Hispanic (Spanish)	−0.82***	_-_1.21***	−0.13	−1.56***	−0.52**	−0.50***	−1.46***	−1.34***
*Vignette 2 (moderate difficulty/intensity)*								
Constant	1.38***	1.84***	0.01	1.43***	−0.43*	0.97***	2.48**	1.88***
Married	0.09	0.01	0.03	−0.15	0.20*	−0.03	0.19	0.04
Male	−0.11	−0.19*	0.01	0.04	0.03	−0.03	−0.40**	0.02
Employed	−0.03	0.03	0.14	0.01	0.06	0.15*	0.15	0.07
More than high school	−0.12	0.03	0.02	0.05	−0.01	0.20**	−0.08	−0.09
Age 18 – 29	0.13	−0.20	0.11	0.14	0.01	0.10	−0.17	−0.32*
Age 30 – 49	−0.06	−0.11	0.12	0.24	0.02	−0.01	−0.22	−0.20
Age 50 – 64	0.01	−0.24	0.10	0.04	0.12	−0.19	−0.18	−0.22
Middle income	0.06	−0.09	−0.06	−0.16	0.09	−0.07	−0.18	−0.05
High income	−0.12	0.00	−0.06	−0.21*	−0.19	−0.18	−0.31	−0.12
Black	0.27	−0.05	0.03	−0.19	0.12	−0.22*	−0.25	−0.16
Hispanic (English)	−0.13	0.12	−0.03	−0.08	0.06	−0.11	−0.22	−0.13
Hispanic (Spanish)	0.02	−0.30**	0.02	−0.35**	0.20	−0.31**	−0.87***	−0.39***

*Notes*: Vignette 3 (highest difficulty/intensity) is the reference vignette.

*: *p* < 0.05;

**: *p* < 0.01;

***: *p* < 0.001.

**Table 6:** Confirmatory Factor Analysis model fit estimates based on the original and anchoring vignette-adjusted scores.

Model	N	CFI	TLI	RMSEA	90% CI of RMSEA
CFA with original self-assessments (full sample)	3,149	0.983	0.948	0.158	(0.138, 0.179)
*Text condition subsample*					
CFA with original self-assessments	1,051	0.986	0.958	0.151	(0.117, 0.189)
CFA with text AV – adjusted self-assessment scores	1,051	0.994	0.982	0.060	(0.026, 0.100)
*Image condition subsample*					
CFA with original self-assessments	2,098	0.981	0.942	0.162	(0.137, 0.188)
CFA with image AV – adjusted self-assessment scores	2,098	0.992	0.977	0.062	(0.038, 0.089)

## Footnotes

1 In designing image vignettes, two different conditions (e.g., male and female) were designed for each domain. Respondents assigned to the image vignette conditions were randomly assigned to the two design conditions. This paper focuses only on the comparison between text and image vignettes, and evaluations on how image vignette design features influence anchoring vignette methodology are discussed elsewhere.

2 Due to the difficulties in recruiting Spanish-speaking Hispanics, Qualtrics collected more respondents for this group in order to meet the targeted number of male Spanish-speaking Hispanics who were 50 and above and had education equal to high school or below.

3 As a sensitivity analysis, we also performed the analysis with 5 minutes and 10 minutes thresholds to identify response time outliers, which gave consistent results.

4 In describing the models, we used the same notation as Grol-Prokopczyk & Carr (2017).

5 The sleep quality score was constructed based on responses to three sleep questions, asking respectively whether and how often respondents 1) have trouble falling asleep, 2) wake up several times at night, and 3) wake up earlier than planned at night and are unable to fall asleep again.

6 The total pain index was constructed following Ray et al. (2009).

7 We examined DIF across race/ethnicity and other socio-demographic groups, including gender, education and marital status. DIF were found across race/ethnicity groups but no other socio-demographic groups.

8 This analysis was also performed when two tie situations were allowed: 1) ties between the first two vignettes (low and moderate intensity) and 2) ties between the last two vignettes (moderate and high intensity). Results of rank order test allowing ties are consistent with Table 3.

9 As a sensitivity analysis, we also fit models combining image and text vignettes in one model for each domain (i.e., treating vignette type as a predictor in the model). Results (shown in Appendix 4) suggests image vignettes perform better in distinguishing the intensity levels of the three vignettes for each domain.

10 We also examined the DIF-adjusting results using HOPIT models. Results are similar for both text and image vignettes. Due to space restrains, results are not shown in this paper and are available upon request.
