# Supplementary material for: Improving Anchoring Vignette Methodology in Health Surveys with Image Vignettes
Source: Methoden Daten Anal. Author manuscript; Available in PMC 2025 Jun 13. (PMC12165436; doi:10.12758/mda.2022.02)
Supplement: 1 [file NIHMS2080921-supplement-1.pdf]

APPENDIX 1

Text and image vignettes used for the web survey for each domain.

Note that in the design of image vignettes, we have two different design conditions per domain. Given that the aim of this paper is to compare text vs. image vignettes, data from different designs of image vignettes are combined in all the analysis. The evaluation the design of features on AV methodology is discussed elsewhere.

Supplemental Table 1 Pain text and image vignettes.

Pain Intensity Level	Text vignette	Image Design One (young adults)	Image Design Two (seniors)
No / Low Pain	Karen has a headache once a month that is relieved after taking a pill. During the headache she can carry on with her day-to-day affairs.	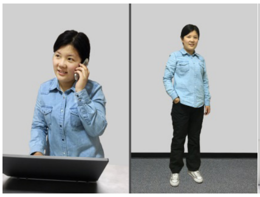	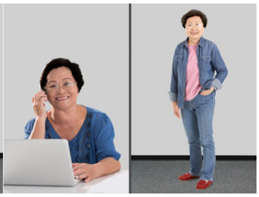
Moderate Pain	Jennifer has pain that radiates down her right arm and wrist during her day at work. This is slightly relieved in the evenings when she is no longer working on her computer.	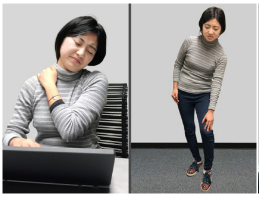	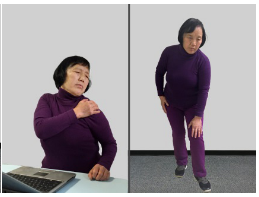
High Pain	Mary has pain in her knees, elbows, wrists and fingers, and the pain is present almost all the time. Although medication helps, she feels uncomfortable when moving around, holding and lifting things.	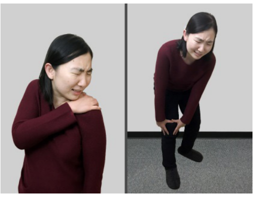	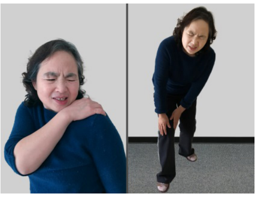

Supplemental Table 2 Sleep text and image vignettes.

Sleep Difficulty Level	Text vignette	Image Design One (female)	Image Design Two (male)
No / Low Difficulty	Sara/Sam falls asleep easily at night, but two nights a week she/he wakes up in the middle of the night and cannot go back to sleep for the rest of the night.	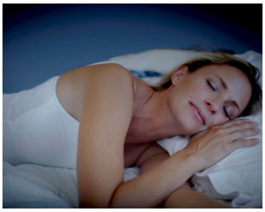	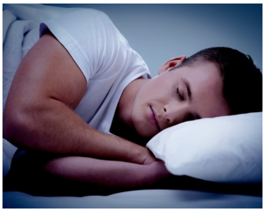
Moderate Difficulty	Susan/Scott wakes up almost once every hour during the night. When she/he wakes up in the night, it takes around 15 minutes for him/her to go back to sleep. In the morning she/he does not feel well-rested.	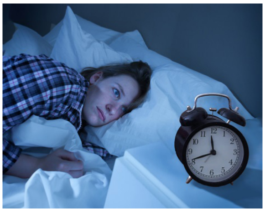	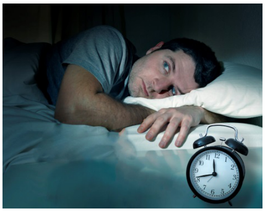
High Difficulty	Patty/Paul takes about two hours every night to fall asleep. She/He wakes up once or twice a night feeling panicked and takes more than one hour to fall asleep again.	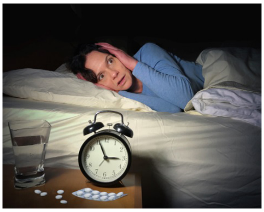	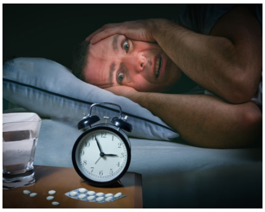

Supplemental Table 3 Mobility text and image vignettes.

Mobility Difficulty Level	Text vignette	Image Design One (optimal weight/fit)	Image Design Two (obese)
No / Low Difficulty	Laura is able to walk distances of up to 200 metres without any problems but feels tired after walking one kilometre or climbing more than one flight of stairs. She has no problems with day-to-day activities, such as carrying food from the market.	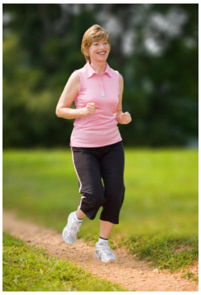	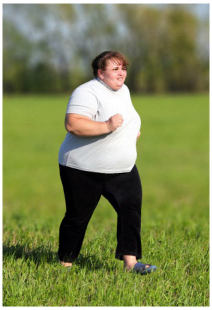
Moderate Difficulty	Sandy does not exercise. She cannot climb stairs or do other physical activities because she is obese. She is able to carry the groceries and do some light household work.	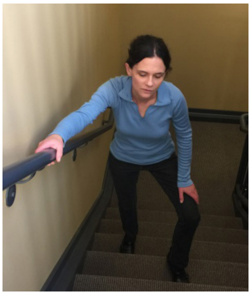	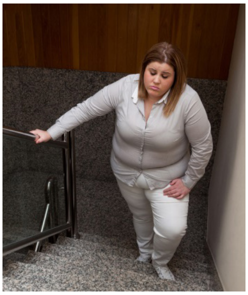
High Difficulty	Lisa has a lot of swelling in her legs due to her health condition. She has to make an effort to walk around her home as her legs feel heavy.	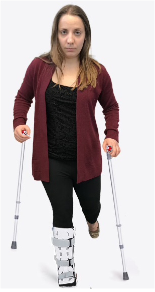	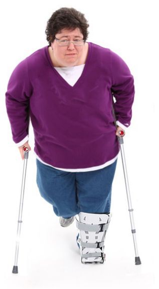

Supplemental Table 4 Affect text and image vignettes.

Depression Level	Text vignette	White	Black	Hispanic
No / Low Depression	Matt enjoys his work and social activities and is generally satisfied with his life. He gets depressed every 3 weeks for a day or two and loses interest in what he usually enjoys but is able to carry on with his day-to-day activities.	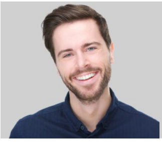	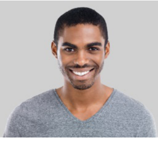	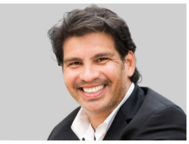
Moderate Depression	David feels nervous and anxious. He worries and thinks negatively about the future but feels better in the company of people or when doing something that really interests him. When he is alone he tends to feel useless and empty.	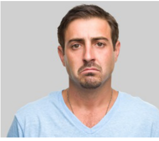	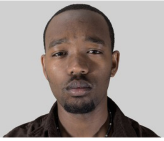	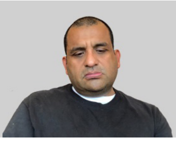
High Depression	Leo feels depressed most of the time. He weeps frequently and feels hopeless about the future. He feels that he has become a burden to others and that he would be better off dead.	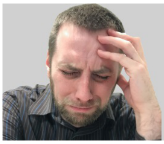	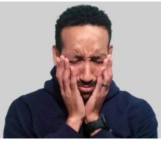	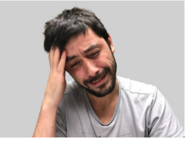

APPENDIX 2

Randomization conditions and assignments and robustness checks for randomization across text and image conditions.

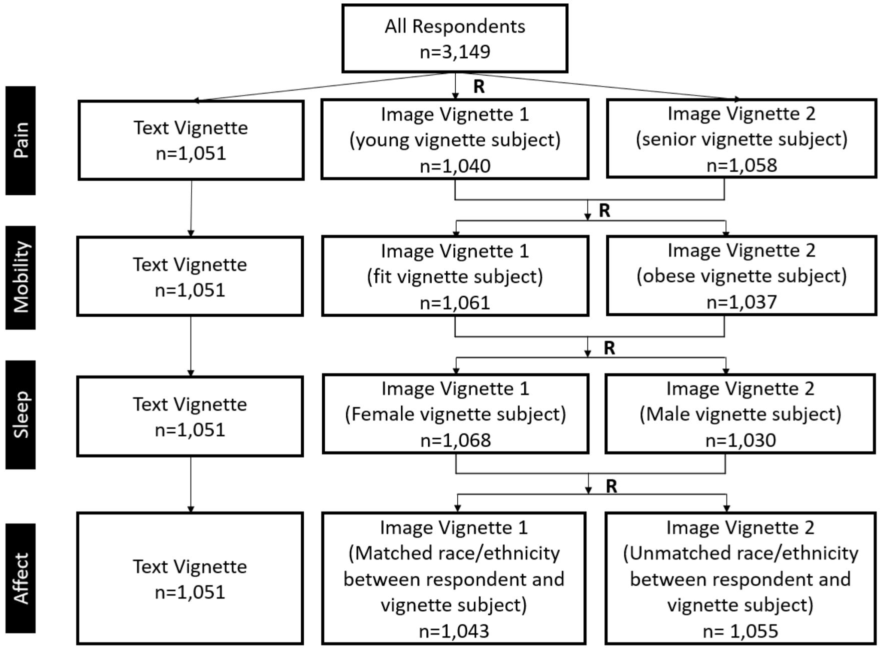

Supplemental Figure 1 Experimental conditions and assignments for each domain. "R" indicates randomization was done.

Supplemental Table 5 Robustness checks for randomization across text and image conditions.

	Text	Image	Chi-square / F statistics
Gender			0.03
Female	52.3	51.9	
Male	47.7	48.1	
Age (mean)	46.9	46.7	0.10
Race			0.47
White	23.8	24.3	
Black	23.8	23.9	
Non-Hispanic White	23.5	24.0	
Non-Hispanic Black	28.9	27.8	
Education			0.01
Below high school	52.2	52.0	
High school and above	47.8	48.0	
Employment status			1.40
Employed	52.6	54.9	
Not employed	47.4	45.1	
Marital status			0.55
Married	50.2	48.8	
Not married	49.8	51.2	
Income			0.84
Low	34.3	34.9	
Middle	42.2	40.6	
High	23.5	24.5	

APPENDIX 3

Distributions of log-transformed response time variable for each domain.

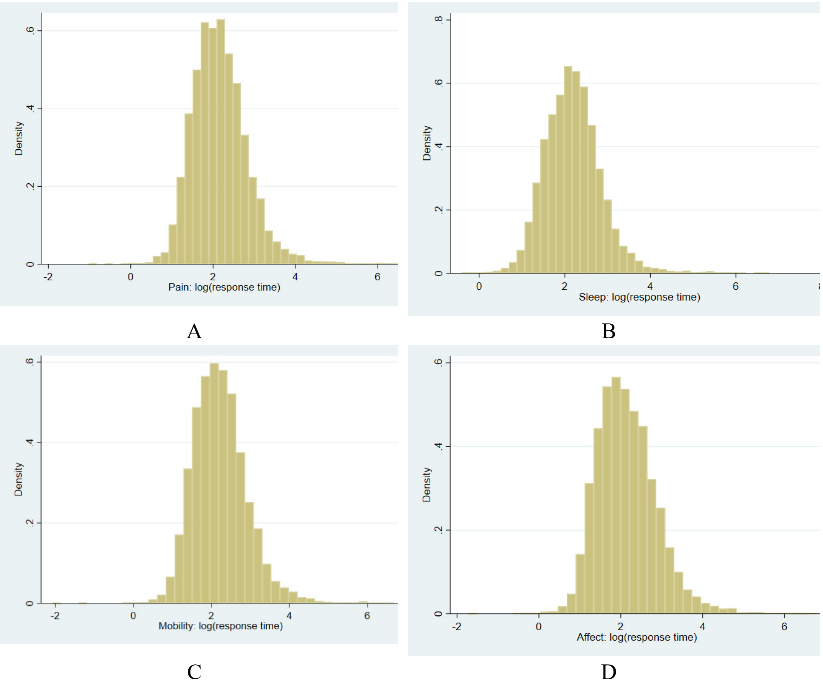

Supplemental Figure 2 Distributions of log-transformed response time variable for each domain.

To formally test the differential response time by vignette types, for each health domain, we fit multilevel logistic regression models with random intercepts. Given that time is right skewed, we used log-transformed time as outcomes (distributions shown in Appendix 3). In the unconditional model (i.e., no predictors in the model) for each domain, log-transformed response time varied significantly across individuals (the intraclass correlation coefficient [ICC] ranges from 0.43 to 0.50, see Supplemental Table 6), justifying the use of multilevel modeling. Supplemental Table 6 shows the results of the final models which include both question level predictors (i.e., image vs. text vignettes) and respondent level predictors (e.g., demographic and socio-economic variables). As shown in Supplemental Table 6, compared to text vignettes, respondents spent significantly less time answering image vignettes. This is true for all four domains. Compared to non-Hispanic White, respondents

of all other three groups spent significantly longer time in answering the vignette questions.

Supplemental Table 6 Multilevel linear regression models predicting log-transformed response time for each health domain.

	Model			
	Pain	Sleep	Mobility	Affect
Image vignettes (ref: Text vignettes)	-0.63***	-0.58***	-0.68***	-0.78***
Age	0.01***	0.01***	0.01***	0.01***
Male (ref: Female)	-0.01	0.00	-0.06**	-0.04*
Above high school education (ref: Below high school)	-0.05**	-0.30	-0.05*	-0.04*
Employed (ref: Not employed)	-0.06**	-0.60**	-0.04*	-0.02
Married (ref: Not married)	-0.05**	-0.45*	-0.04	-0.06**
Respondent Groups (Ref: Non-Hispanic White)				
Non-Hispanic Black	0.18***	0.20***	0.18***	0.18***
Hispanics English	0.06*	0.07**	0.09**	0.07**
Hispanics Spanish	0.16***	0.16***	0.14***	0.17***
ICC (95% confidence interval)	0.50 (0.48, 0.52)	0.45 (0.43, 0.47)	0.49 (0.47, 0.51)	0.43 (0.41, 0.45)

*: $p < 0.05$; **: $p < 0.01$; ***: $p < 0.001$.

APPENDIX 4

Model results for evaluating VE test for each domain (with both image and text vignettes combined for analysis).

Supplemental Table 7 Predictors for perceived vignette locations on the latent health spectrum.

	Pain	Sleep	Mobility	Affect
Vignette 1 (no/mild difficulty/intensity)				
Constant	3.39***	1.74***	1.90***	4.10***
Image	1.59***	2.84***	0.17**	1.07***
Married	0.21*	-0.07	0.12	0.15
Male	-0.43***	-0.23**	-0.14*	-0.24**
Employed	-0.02	0.15	0.10	0.12
More than high school	-0.03	0.11	0.12	-0.10
Age 18 - 29	-0.20	0.02	-0.12	-0.50**
Age 30 - 49	-0.21	0.10	-0.09	-0.40**
Age 50 - 64	-0.27*	0.09	-0.05	-0.39**
Middle income	-0.14	-0.11	0.00	-0.19*
High income	-0.05	-0.19	-0.31***	-0.46***
Black	-0.08	-0.13	-0.15	-0.44***
Hispanic (English)	-0.17	-0.14	-0.04	-0.17
Hispanic (Spanish)	-0.89***	-0.55***	-0.48***	-1.13***
Vignette 2 (moderate difficulty/intensity)				
Constant	1.58***	0.20	-0.14	2.21***
Image	0.16*	1.03***	0.98***	-0.27***
Married	0.01	-0.07	0.06	0.06
Male	-0.16*	0.00	0.00	-0.08
Employed	0.00	0.05	0.10	0.09
More than high school	-0.04	0.04	0.12*	-0.10
Age 18 - 29	-0.06	0.15	0.07	-0.33**
Age 30 - 49	-0.12	0.18	-0.01	-0.26*
Age 50 - 64	-0.15	0.07	-0.08	-0.24*
Middle income	-0.04	-0.08	-0.03	-0.10
High income	-0.05	-0.14	-0.18*	-0.18*
Black	0.05	-0.07	-0.12	-0.11
Hispanic (English)	0.00	-0.05	-0.05	-0.11
Hispanic (Spanish)	-0.15	-0.19*	-0.15*	-0.46***

Notes: Vignette 3 (highest difficulty/intensity) is the reference vignette. *: $p < 0.05$; **: $p < 0.01$; ***: $p < 0.001$.
